# Investigating associations between personality and problematic social media use by considering trait and state FoMO as mediators

**DOI:** 10.1186/s40359-026-05527-w

**Published:** 2026-11-02

**Authors:** Yao Wang, Christopher Kannen, Zhiying Zhao, Brian J. Hall, Christian Montag

**Affiliations:** 1 Institute of Artificial Intelligence and Brain Sciences, University of Macau, Macau SAR, China; 2 Institute of Psychology and Education, Ulm University, Ulm, Germany; 3 Center for Global Health Equity, NYU Shanghai, Shanghai, China; 4 Department of Computer Science, University of Macau, Macau SAR, China; 5 Department of Psychology, University of Macau, Macau SAR, China

**Keywords:** Personality, Fear of missing out, Problematic social media use, Social media addiction

## Abstract

High Neuroticism and low Conscientiousness robustly correlate with both Problematic Social Media Use (PSMU) and Fear of Missing Out (FoMO). However studies investigating these concepts simultaneously are relatively rare, and few have examined the associations between the personality traits and PSMU by investigating both trait and state FoMO as potential mediators. In a sample of 576 German-speaking participants (of whom *n* = 94 were aged 13–17), we observed that higher Neuroticism and lower Conscientiousness were associated with higher PSMU. Mediation results indicated that Neuroticism was indirectly associated with PSMU via both state FoMO (β = 0.10, *b* = 0.63, Proportion Mediated [PM] = 27.3%, 95% CI [0.36, 0.91]) and trait FoMO (β = 0.05, b = 0.33, PM = 14.3%, 95% CI [0.13, 0.53], *p* < .001). Conversely, for Conscientiousness, only state FoMO emerged as a significant mediator (β = -0.06, *b* = -0.48, PM = 33.8%, 95% CI [-0.81, -0.16], *p* = .002); the indirect pathway via trait FoMO was non-significant (β = -0.02, *b* = -0.12, 95% CI [-0.28, 0.02], *p* = .09). These findings suggest that state FoMO, which reflects online-specific anxiety, is a more consistent factor linking personality traits to PSMU than general trait FoMO. This distinction suggests that state FoMO could be considered a potential target for practical interventions, as it is more situational and might be easier to change than personality traits.

**Supplementary Information:**

The online version contains supplementary material available at https://doi.org/10.1186/s40359-026-05527-w.

## Introduction

The widespread adoption of social media has provided users with convenient channels for communication and information access, but it is also associated with risks of overuse and the addiction concept is discussed in this context [1–3]. An expanding body of research highlights the risks of overuse, which can culminate in a behavioral addiction phenomenon often coined as Problematic Social Media Use (PSMU). PSMU has become an important research topic in the psychological sciences [4]. Given that billions of individuals engage with social media daily, exploring the underlying psychological mechanisms linked to the transition from normative use to problematic (addiction-like) behavior is of critical scientific and clinical importance [5].

### Personality, problematic social media use and the I-PACE framework

The Interaction of Person-Affect-Cognition-Execution (I-PACE) model [6] provides a theoretical framework for understanding the development of PSMU. The model posits that predisposing individual characteristics (Person, P) do not directly lead to addictive behaviors, but relate to problematic usage patterns through specific affective (Affect, A) and cognitive (Cognition, C) pathways. Based on this framework and consistent meta-analytic evidence [7], high Neuroticism and low Conscientiousness have been identified as robust risk factors for Internet Use Disorders [8–10]. Accordingly, our study focused on these two traits as the primary predictors for the mediation analysis.

A relevant psychological mechanism that fits within the “Affect” and “Cognition” components of the I-PACE model is the Fear of Missing Out (FoMO) [11]. FoMO describes a chronic concern that others are having rewarding experiences in one’s absence, a state strongly associated with social media engagement [11]. Meta-analytic findings show a robust positive correlation between FoMO and PSMU [12], suggesting that PSMU may be related to an urge to remain online as a way to cope with negative feelings [12, 13]. Recent theoretical advancements propose a critical distinction within FoMO, which conceptualizes FoMO as a two-dimensional construct, comprising trait FoMO (a stable, dispositional tendency to experience this fear in general life contexts) and state FoMO (a transient, situation-specific fear triggered by and experienced within online environments) [14, 15]. Within the I-PACE framework, trait FoMO can be seen as a distal cognitive component, while state FoMO represents a proximal affective reaction to online cues. According to the model, proximal mediators often exhibit stronger associations with the outcome than distal variables as they are conceptually closer to the behavioral execution phase [6, 16].

### Differentiating trait versus state FoMO in the mediation pathway

The question of how personality traits, FoMO, and PSMU are linked is an emerging research focus. As suggested by the I-PACE model, FoMO may mediate the relationship between personality traits and PSMU [16]. Ruyandy and Kartasasmita observed that FoMO mediated the relationship between Big Five personality traits such as Neuroticism and Conscientiousness, and Problematic Internet Use (PIU) [17], which is robustly linked to PSMU [18]. Further work observed that the association between Neuroticism and problematic WeChat use is also mediated by FoMO, hence suggesting such a mediation within the area of PSMU [19]. Although these results are promising, most previous studies have treated FoMO as a unitary construct, leaving it unclear how the relative importance of trait versus state FoMO differs in these mediation pathways. Importantly (and as mentioned), trait and state FoMO are qualitatively distinct. The former represents a stable disposition, while the latter represents an online-specific anxiety [15]. Psychometric validation confirms their discriminant validity, justifying their evaluation as separate mediators despite being moderately correlated [15]. Therefore, distinguishing between them is critical for revealing the specific psychological mechanisms underlying PSMU.

### Hypotheses

This study aims to fill this gap by exploring the mediating effects of trait and state FoMO between the Big Five personality traits and PSMU, and by comparing the strength of their roles, to reveal potential psychological mechanisms underlying the personality-PSMU-link. Based on previous research, we hypothesized that (a) both higher Neuroticism and lower Conscientiousness will be consistently associated with higher PSMU [20]; (b) FoMO will significantly mediate the relationship between Neuroticism/Conscientiousness and PSMU; and (c) as an exploratory directional hypothesis, state FoMO, as a proximal situational trigger, will exhibit a stronger mediating effect in these relationships than trait FoMO.

## Method

### Participants

The present study was subsumed within a larger study on WhatsApp use. This larger study stems from the overarching research project, in which Kannen et al., using another data set, investigated willingness to pay for social media [21].

For the present work of relevance, a sample of *N* = 784 was recruited and the inclusion criteria were everyone being 13 years or older using WhatsApp. Participants being younger than 18 years (but 13 years or older) were required to discuss the study with their parents or guardians prior to participation; upon parental or guardian consent, these minors confirmed via a tick-box that the discussion had occurred and that both the parents/guardians and the minor agreed to the minor’s involvement in the study. After providing informed e-consent, participants filled in a battery of questionnaires. The study was approved by the local ethics committee (Ulm University; Number 93/20). Research was carried out in compliance with the Declaration of Helsinki.

### Data cleaning

A total of 208 participants were removed for not meeting the study inclusion criteria (6 participants with self-reported age under 13 years, 13 who did not use WhatsApp, 126 who stated they did not use social media, and 63 participants who did not use messenger apps). We also excluded participants not using messenger, because messenger can be seen as a social media product [22]. As described above, minor-aged participants (hence being 13 years or older, but under 18 years) had obtained consented from their parents. The final sample of this study is *N* = 576 (175 males, age-range: 13 to 69; 23.95 ± 10.27; and 401 females, age-range: 13 to 65; 24.33 ± 8.44; age under 18 years *n* = 94, 18 years or above *n* = 482). A final note: During the data cleaning process we observed five participants with longer same consecutive answers (often a neutral answer). Given that these could be true answers, we did not take these participants out. But we checked if this would change the overall data pattern in a meaningful way. This was not the case.

### Questionnaires

#### Big Five Inventory

The German version of the BFI was used to assess the Big Five of Personality consisting of 45 items [23] and was answered via a five-point Likert scale. Higher scores of each dimension indicate a higher expression of each personality trait. The internal consistencies (Cronbach’s alpha) were good to excellent: Openness to Experience (α = 0.81), Conscientiousness (α = 0.82), Extraversion (α = 0.86), Agreeableness (α = 0.75) and Neuroticism (α = 0.84).

#### Fear of Missing Out scale (FoMO scale)

The FoMO scale is used to assess both state and trait aspects of FoMO. The scale includes 12 items, with 5 items measuring trait FoMO and 7 items measuring state FoMO. For example, one item assessing trait FoMO goes as follows “I fear others have more rewarding experiences than me”. An exemplary item for state FoMO is formulated as “I am continuously online in order not to miss out on anything”. Participants rate each item on a five-point Likert scale (ranging from *1 = strongly disagree to 5 = strongly agree)*, with higher scores indicating higher levels of the respective FoMO measure (trait vs. state). The German version was used as provided by Elisa Wegmann [15]. The internal consistencies calculated from the present dataset were α = 0.78 for trait FoMO and α = 0.75 for state FoMO respectively.

####  Social Networking Sites-Addiction Test (SNS-AT)

PSMU was assessed using an addiction framework, employing the German version by Montag et al. [24]. The Social Networking Sites – Addiction Test consists of six items, which are summed. Higher scores indicate more PSMU tendencies. The internal consistencies calculated from this dataset were α = 0.80.

### Data analysis

The data analyses were conducted with jamovi 2.6.26.0 (https://www.jamovi.org). First, descriptive statistics (mean, standard deviation, skewness, and kurtosis) were examined. While more conservative thresholds exist, we made an analytic decision to use absolute values of skewness and kurtosis less than 2 to indicate a normal distribution [25], to determine whether parametric or non-parametric tests should be used for subsequent analyses, thereby ensuring the robustness of the results. Additionally, independent samples t-tests were performed to test for differences in these variables between genders (male and female), to determine if gender was a potential confounding variable. Furthermore, to verify the two-dimensional structure of the FoMO scale, a Confirmatory Factor Analysis (CFA) was conducted. The fit indices for the two-factor model did not meet conventional thresholds (CFI = 0.809, TLI = 0.762, RMSEA = 0.127). However, as we employed the established German version of the scale [15], the original item structure was retained to preserve theoretical integrity and ensure comparability with prior literature. Moreover, a unidimensional alternative model yielded a substantially poorer fit (CFI = 0.656, TLI = 0.579, RMSEA = 0.168), supporting the retention of the two dimensions. Regarding discriminant validity, although the Fornell-Larcker condition was met ($\:\sqrt{AVE}$ = 0.574 > *r* = 0.451), the convergent validity for the state FoMO subscale was below the standard threshold (AVE = 0.329). Given these model fit characteristics, the psychometric properties of the scale in the current sample should be interpreted with appropriate caution. Detailed CFA results are provided in the Supplementary Information (Supplementary Table 3).

We tested the correlations of all Big Five traits and PSMU. However, based on the I-PACE model and previous studies, we tested mediation models only for Neuroticism and Conscientiousness as predictors (in line with our hypotheses).

Consistent with the theoretical framework, parallel mediation models were constructed using Neuroticism and Conscientiousness as “predictors” (no causality implied), with SNS-AT (PSMU) as the outcome. Trait and state FoMO were entered simultaneously as mediators. This approach estimates the specific indirect effect of each FoMO dimension while controlling for the other. Analyses were performed in jamovi (jAMM module) using a bootstrapping method (5000 resamples, 95% CIs) to estimate direct and indirect associations. The current sample size (*N* = 576) provides robust statistical power for the analyses. According to established guidelines for bootstrapped mediation, a sample of approximately 460 is sufficient to detect small-to-medium indirect effects with 0.80 power [26]. To formally compare the relative magnitude of the two mediating pathways, the statistical contrast between the specific indirect effects (state FoMO minus trait FoMO) was computed and evaluated via bootstrapped confidence intervals using a custom MATLAB script.

For clarity, the models reported in the main text include only the primary variables. Finally, to address psychometric and developmental considerations regarding the minor subsample, a sensitivity analysis was performed by re-estimating the mediation models exclusively on the adult cohort (age ≥ 18; *n* = 482). The detailed outcomes of this developmental sensitivity analysis, alongside additional models adjusting for age and gender covariates, are comprehensively documented in the Supplementary Information (Supplementary Tables 1 and 2).

## Results

### Descriptive statistics and bivariate correlations

Table 1 shows the descriptive statistics for age, trait FoMO, state FoMO, and SNS-AT (PSMU). The age distribution exhibited significant skewness (see Supplementary Information for the detailed frequency distribution and histogram); however, age did not significantly differ between genders (see Table 2). Trait FoMO, state FoMO, and SNS-AT scores were approximately normally distributed, supporting the use of parametric tests (see Table 1). While no significant gender differences were found for trait or state FoMO, a significant difference emerged for SNS-AT, with males scoring significantly lower than females (t_(574)_ = −2.07, p = 0.04, at least with the parametric test) (Table 2).

**Table 1:** Descriptive statistics for the male and female subsample

	Gender	N	Missing	Mean	Median	SD	Skewness	SE	Kurtosis	SE
**Age**	**Male**	175	0	23.95	22	10.27	1.65	0.18	3.28	0.37
	**Female**	401	0	24.33	22	8.44	2.25	0.12	6.04	0.24
**Trait FoMO (Mean)**	**Male**	175	0	3.12	3.20	0.79	-0.02	0.18	-0.44	0.37
	**Female**	401	0	3.25	3.20	0.80	-0.30	0.12	-0.20	0.24
**State FoMO (Mean)**	**Male**	175	0	2.60	2.71	0.83	0.07	0.18	-0.42	0.37
	**Female**	401	0	2.54	2.43	0.82	0.23	0.12	-0.63	0.24
**SNS-AT**	**Male**	175	0	14.79	15	4.89	0.01	0.18	-0.56	0.37
	**Female**	401	0	15.73	16	5.03	0.06	0.12	-0.45	0.24

Note for Mean column: FoMO scores reflect item means (range 1–5), whereas SNS-AT reflects sum scores

**Table 2:** Independent samples t-tests/Mann-Whitney U tests investigating gender effects in the realm of the variables presented

		Statistic	df	*p*		Effect Size
**Age**	**Student's t**	-0.46ᵃ	574	0.64	Cohen's d	-0.04
	**Mann-Whitney U**	31,438		0.05	Rank biserial correlation	-0.10
**Trait FoMO**	**Student’s t**	-1.72	574	0.09	Cohen’s d	-0.16
	**Mann-Whitney U**	31,585		0.06	Rank biserial correlation	-0.10
**State FoMO**	**Student’s t**	0.81	574	0.42	Cohen’s d	0.07
	**Mann-Whitney U**	33,382		0.35	Rank biserial correlation	0.05
**SNS-AT**	**Student’s t**	-2.07	574	0.04	Cohen’s d	-0.19
	**Mann-Whitney U**	31,651		0.06	Rank biserial correlation	-0.10

Note. Hₐ µ_male_ ≠ µ_female_

ᵃ Levene’s test is significant (*p* < .05), suggesting a violation of the assumption of equal variances

Table 3 presents the results of the correlation analyses between the Big Five personality traits, trait FoMO, state FoMO, and SNS-AT. Trait and state FoMO were moderately correlated (*r* = .45, *p* < .001), sharing approximately 20.3% of their variance. This supports their conceptual relatedness under the FoMO construct while confirming adequate discriminant validity to function as distinct parallel mediators. The results show that SNS-AT has a strong positive correlation with state FoMO (*r* = .59, *p* < .001) while the correlation with trait FoMO is a bit weaker but still in the moderate effect size range (*r* = .44, *p* < .001). For the Big Five traits, Extraversion, Agreeableness, and Openness exhibited weak or non-significant associations with both FoMO dimensions and SNS-AT (nevertheless we mention the inverse association between Extraversion/trait FoMO with SNS-AT: both r = -.15, p < .001). Therefore, to preserve model parsimony (and because of the hypotheses), these three traits were excluded from the primary mediation models.

**Table 3:** Correlation Matrix

		Extraversion	Neuroticism	Agreeableness	Openness	Conscientiousness	Trait FoMO	State FoMO	SNS-AT
**Extraversion**	**Pearson’s r**	—							
	**p-value**	—							
**Neuroticism**	**Pearson’s r**	-0.35***	—						
	**p-value**	< 0.001	—						
**Agreeableness**	**Pearson’s r**	0.11**	-0.24***	—					
	**p-value**	0.01	< 0.001	—					
**Openness**	**Pearson’s r**	0.24***	-0.05	0.24***	—				
	**p-value**	< 0.001	0.24	< 0.001	—				
**Conscientiousness**	**Pearson’s r**	0.19***	-0.23***	0.21***	0.08	—			
	**p-value**	< 0.001	< 0.001	< 0.001	0.06	—			
**Trait FoMO**	**Pearson’s r**	-0.15***	0.36***	-0.06	-0.08	-0.07	—		
	**p-value**	< 0.001	< 0.001	0.12	0.07	0.08	—		
**State FoMO**	**Pearson’s r**	-0.05	0.21***	-0.09*	-0.11**	-0.14**	0.45***	—	
	**p-value**	0.25	< 0.001	0.03	0.01	0.001	< 0.001	—	
**SNS-AT**	**Pearson’s r**	-0.15***	0.36***	-0.02	-0.06	-0.19***	0.44***	0.59***	—
	**p-value**	< 0.001	< 0.001	0.60	0.16	< 0.001	< 0.001	< 0.001	—

Note. * *p* < .05, ** *p* < .01, *** *p* < .001

Both Neuroticism and Conscientiousness were significantly correlated with SNS-AT (*r* = .36, *p* < .001 and *r* = -.19, *p* < .001, respectively). Importantly, Neuroticism was positively correlated with both trait (*r* = .36, *p* < .001) and state FoMO (*r* = .21, *p* < .001). In contrast, Conscientiousness was significantly correlated only with state FoMO (*r* = -.14, *p* = .001), and showed no significant correlation with trait FoMO (*r* = -.07, *p* = .08). This specific correlation pattern serves as meaningful preliminary evidence foreshadowing the mediation results, highlighting state FoMO as the predominant mechanism linking Conscientiousness to PSMU. Further sensitivity analysis excluding participants under 18 years of age (*n* = 94) confirmed that all primary correlational associations remained robust (see Supplementary Tables 5, 6 and 7).

### The mediating roles of trait and state FoMO

In accordance with the established theoretical framework, mediation models were constructed specifying Neuroticism and Conscientiousness as independent predictor variables (no causality implied), with trait and state FoMO acting as parallel mediators and SNS-AT being the outcome variable. Despite the previously noted gender effect on SNS-AT, these primary models are presented unadjusted. Covariate-adjusted models explicitly controlling for gender and age are detailed in the Supplementary Information (Supplementary Tables 1 and 2) and summarized at the end of this section.

As detailed in Table 4, Neuroticism exhibited a significant indirect association with SNS-AT through both state FoMO (β = 0.10,* b* = 0.63, PM = 27.3%, 95% CI [0.36, 0.91], *p* < .001) and trait FoMO (β = 0.05, *b* = 0.33, PM = 14.3%, 95% standardized CI [0.13, 0.53], *p* < .001). Although the mediating effect of state FoMO was numerically larger, a formal bootstrap contrast analysis indicated that the difference between the two indirect pathways did not reach statistical significance (Contrast estimate = 0.30, 95% CI [-0.04, 0.68]). The direct association between Neuroticism and SNS-AT remained significant (β = 0.22, *p* < .001), indicating partial mediation.

**Table 4:** Parallel mediation model with the variables Neuroticism (predictor), State FoMO & Trait FoMO (mediators) and SNS-AT (outcome)

				95% CI				
Type	Effect	Estimate	SE	Lower	Upper	β	z	*p*
**Indirect**	**Neuroticism ⇒ Trait FoMO ⇒ SNS-AT**	0.33	0.09	0.13	0.53	0.05	3.57	< 0.001
	**Neuroticism ⇒ State FoMO ⇒ SNS-AT**	0.63	0.13	0.36	0.91	0.10	4.73	< 0.001
**Component**	**Neuroticism ⇒ Trait FoMO**	1.80	0.20	1.42	2.17	0.36	9.13	< 0.001
	**Trait FoMO ⇒ SNS-AT**	0.18	0.05	0.07	0.28	0.14	3.88	< 0.001
	**Neuroticism ⇒ State FoMO**	1.49	0.30	0.89	2.08	0.21	5.04	< 0.001
	**State FoMO ⇒ SNS-AT**	0.42	0.03	0.35	0.49	0.48	13.60	< 0.001
**Direct**	**Neuroticism ⇒ SNS-AT**	1.36	0.21	0.92	1.80	0.22	6.32	< 0.001
**Total**	**Neuroticism ⇒ SNS-AT**	2.31	0.25	1.82	2.81	0.36	9.37	< 0.001

Note. Betas are completely standardized effect sizes

Note. Confidence intervals computed with method: Bootstrap percentiles

**Table 5:** Parallel mediation model with the variables Conscientiousness (predictor), State FOMO & Trait FOMO (mediators) and SNS-AT (outcome)

				95% CI				
Type	Effect	Estimate	SE	Lower	Upper	β	z	*p*
**Indirect**	**Conscientiousness ⇒ Trait FoMO ⇒ SNS-AT**	-0.12	0.07	-0.28	0.02	-0.02	-1.70	0.09
	**Conscientiousness ⇒ State FoMO ⇒ SNS-AT**	-0.48	0.15	-0.81	-0.16	-0.06	-3.17	0.002
**Component**	**Conscientiousness ⇒ Trait FoMO**	-0.44	0.25	-0.94	0.10	-0.07	-1.78	0.08
	**Trait FoMO ⇒ SNS-AT**	0.27	0.05	0.16	0.38	0.21	5.87	< 0.001
	**Conscientiousness ⇒ State FoMO**	-1.15	0.35	-1.90	-0.39	-0.14	-3.27	0.001
	**State FoMO ⇒ SNS-AT**	0.42	0.03	0.35	0.49	0.48	13.16	< 0.001
**Direct**	**Conscientiousness ⇒ SNS-AT**	-0.83	0.24	-1.32	-0.33	-0.11	-3.40	< 0.001
**Total**	**Conscientiousness ⇒ SNS-AT**	-1.42	0.30	-2.11	-0.77	-0.19	-4.68	< 0.001

Note. Betas are completely standardized effect sizes

Note. Confidence intervals computed with method: Bootstrap percentiles

Conversely, in the Conscientiousness model (Table 5), only the indirect pathway through state FoMO reached statistical significance (β = -0.06, *b* = -0.48, PM = 33.8%, 95% CI [-0.81, -0.16], *p* = .002). The indirect effect via trait FoMO was not significant (β = -0.02, *b* = -0.12, 95% CI [-0.28, 0.02], *p* = .09). The direct effect of Conscientiousness on SNS-AT also remained significant (β = -0.11, *p* < .001).

Furthermore, the parallel mediation models were re-estimated incorporating age and gender as covariates. The inclusion of these covariates did not alter the primary results. For Neuroticism, both state FoMO (β = 0.10, *p* < .001) and trait FoMO (β = 0.04, *p* < .001) remained significant mediators. For Conscientiousness, state FoMO maintained its significance as a robust mediator (β = -0.06, *p* = .003), whereas the indirect pathway via trait FoMO was not significant (β = -0.01, *p* = .10). The results indicate that state FoMO reached statistical significance as a mediator across both personality pathways, whereas trait FoMO did not. However, as evidenced by the non-significant bootstrap contrast test, the difference in the magnitude of the indirect effects within the Neuroticism pathway was not statistically confirmed.

Finally, the sensitivity analysis conducted on the adult-only cohort (*n* = 482) yielded identical mediational patterns for Neuroticism. However, a notable developmental divergence emerged in the Conscientiousness model that the indirect pathway via trait FoMO, which was non-significant in the full sample, reached statistical significance among adults. The state FoMO pathway remained robustly significant across all analyses. Detailed statistical parameters for this age-restricted sensitivity analysis are provided in the Supplementary Information (see Supplementary Tables 5, 6 and 7).

## Discussion

This study aimed to explore the psychological mechanisms underlying PSMU by investigating the relationships between Big Five personality traits, FoMO and PSMU. Overall, the results corroborated our theoretically driven hypotheses. First, regarding the direct associations, higher Neuroticism and lower Conscientiousness, as well as both dimensions of FoMO, were significantly associated with higher PSMU. Second, regarding the psychological mechanisms, our mediation analyses revealed two distinct pathways to PSMU: the association between Neuroticism and PSMU was mediated by both trait and state FoMO, whereas the link between low Conscientiousness and PSMU was uniquely mediated by state FoMO. It is worth noting that for Neuroticism, the indirect effect of state FoMO was not statistically stronger than that of trait FoMO (as evidenced by the non-significant bootstrap contrast test). Therefore, rather than having a stronger effect size, state FoMO’s key role lies in its consistency: it acts as a significant mediator across different personality profiles. Together, these findings highlight the necessity of differentiating between general dispositional anxiety (trait FoMO) and online-specific anxiety (state FoMO) when understanding the development of PSMU.

To elucidate the behavioral pathways leading to PSMU, the present study initially evaluated its direct associations with personality traits and FoMO. Consistent with established meta-analytic evidence [7, 27, 28], the results demonstrated that higher Neuroticism and lower Conscientiousness were significantly associated with increased PSMU. One interpretation for the robust link between Neuroticism and PSMU can be understood through the lens of emotion regulation and the compensation-seeking hypothesis. Individuals high in Neuroticism frequently experience emotional instability and psychological distress; consequently, they are more likely to turn to the digital environment as a maladaptive coping strategy to escape from reality or seek online reassurance [29]. Conversely, the negative association between Conscientiousness and PSMU is primarily driven by individual differences in top-down inhibitory control [16, 30]. Conscientious individuals generally possess strong self-regulation skills that buffer against excessive digital engagement. In contrast, those low in Conscientiousness struggle to delay gratification and resist immediate digital rewards, making them highly susceptible to the habitual checking behaviors elicited by social media cues. Furthermore, although not explicitly hypothesized, our analyses indicated a negative association between Extraversion and trait FoMO as well as PSMU. This suggests that introverted individuals might tend to exhibit higher levels of a stable, trait-like expression of FoMO alongside problematic social media use. This pattern aligns with the social compensation hypothesis, wherein individuals who find offline face-to-face interactions challenging may disproportionately rely on digital environments to fulfill their social needs, a behavioral pattern that corresponds to greater vulnerability to problematic use [31, 32]. Finally, both dimensions of FoMO correlated positively with PSMU. Notably, SNS-AT (an indicator of PSMU) correlated more strongly with state FoMO than with trait FoMO [33]. This supports the theoretical distinction that while a general dispositional worry (trait FoMO) creates a psychological vulnerability, it is the immediate, context-specific anxiety to stay connected online (state FoMO) that most directly drives addiction-like social media use [15]. Of note, the overall Neuroticism-PSMU link seems to be more relevant than the Conscientiousness-PSMU link when considering the effect sizes.

### The distinct mediating functions of trait and state FoMO

The finding that state FoMO correlates more strongly with PSMU than with trait FoMO suggests that immediate, context-specific anxiety is more closely linked to PSMU tendencies than general FoMO in the context of online areas [15]. State FoMO is often tied to real-time social media cues (like notifications), reflecting users’ instant anxiety to stay connected online and potentially leading to patterns of repeatedly checking platforms. Our mediation analyses further specified these roles. Within the I-PACE framework, trait FoMO represents a distal cognitive predisposition, while state FoMO acts as a proximal trigger for checking behavior [16]. Our findings indicated a divergent mediation profile for Neuroticism and Conscientiousness. This aligns with and refines previous research. Similar to Ruyandy and Kartasasmita [17] and Sindermann et al. [19], we observed that FoMO serves as a significant mediator linking these two personality traits to problematic social media use. However, prior studies have typically conceptualized FoMO as a unitary construct, implicitly suggesting that different personality traits relate to PSMU through the same general mechanism. By distinguishing between trait and state FoMO, our results extend these findings to reveal trait-specific pathways: the association for Neuroticism involves both dispositional worry and situational anxiety, whereas the association for low Conscientiousness might be more exclusively tied to the immediate, situational urge to stay online.

The association between Neuroticism and PSMU was mediated by both trait and state FoMO. From a theoretical standpoint, this dual pathway reflects multifaceted psychological mechanisms rather than a single underlying process. High Neuroticism has been consistently linked to a generalized fear of social exclusion (trait FoMO) [20, 34], which often co-occurs with an increased affective reactivity to immediate online cues (state FoMO) [15, 35]. This pattern aligns with the reassurance-seeking model of problematic internet use [36, 37]. It posits that individuals with higher Neuroticism scores may use social media as a compensatory strategy to alleviate chronic psychological distress and manage acute situational anxieties associated with the digital environment [38, 39]. Within the I-PACE framework, these results suggest that the vulnerability associated with Neuroticism corresponds to two interrelated components: a stable cognitive tendency to worry about missing out (trait FoMO) and an acute affective reaction to digital cues (state FoMO). Consequently, their social media engagement may reflect a behavioral pattern aimed at managing both chronic emotional distress and the immediate anxiety of remaining connected. Conversely, the statistical mediation for Conscientiousness involved only the proximal mechanism. The indirect pathway via trait FoMO was non-significant, indicating that the association between lower Conscientiousness and PSMU in the current sample was not primarily explained by a generalized dispositional worry about missing out. Instead, the association was specifically mediated by state FoMO. This distinct pattern is consistent with the core psychological features of low Conscientiousness, which may be characterized primarily by deficits in top-down inhibitory control rather than persistent emotional distress [40, 41]. Individuals scoring lower in Conscientiousness do not necessarily exhibit a chronic cognitive schema of social exclusion (trait FoMO). Instead, when encountering environmental triggers (e.g., smartphone notifications), they may experience difficulties in utilizing executive resources to inhibit the impulse to check social media, thereby showing a stronger susceptibility to acute situational arousal (state FoMO) [42, 43]. In summary, while the association between Neuroticism and PSMU is characterized by a combination of internal emotional distress and reactivity to external cues, the link between low Conscientiousness and PSMU is better understood as a vulnerability to immediate online triggers stemming from reduced behavioral inhibition.

Finally, the sensitivity analysis regarding age groups identified a developmental difference (see Supplementary Information). The indirect pathway from Conscientiousness to PSMU via trait FoMO was significant only in the adult subsample. Personality traits generally show lower stability during adolescence and consolidate over time [44]. This result suggests that the link between low Conscientiousness and stable trait FoMO fully matures in adulthood. Meanwhile, state FoMO serves as a consistent mediator across age groups. A possible explanation is that as individuals grow older, repeated failures in self-regulation (due to low Conscientiousness) might gradually lead to a stable worry about missing social opportunities (trait FoMO). For adolescents, low Conscientiousness may simply reflect a developing brain struggling to resist smartphone notifications (state FoMO). In adults, however, this lack of behavioral control might eventually become linked to broader worries about their social participation. This said, our observations need to be replicated in the future, being backed up by neuroscientific data and our interpretation should be seen as preliminary (in particular also regarding the causality assumptions).

### Implications for theory and intervention

The consistent role of state FoMO across our models offers practical directions for interventions. Personality traits are generally stable and difficult to change in the short term. In contrast, state FoMO is highly situational and easier to target for behavioral change [45]. For individuals with low Conscientiousness, interventions can focus on environmental nudges rather than personality restructuring. Preliminary evidence from a single short-term intervention suggests some potential for modest trait-level change [46], though the evidence base remains limited and the long-term efficacy of such approaches is untested. Environmental nudges could lie within reducing social media notification frequencies, delaying information display, or enhancing users’ self-awareness of social cues, which could help alleviate the experience of FoMO [9]. Importantly, FoMO only partially mediated the relationship between Neuroticism and PSMU. The remaining direct effect suggests that interventions targeting only FoMO may not be sufficient for individuals high in Neuroticism. This finding points to the involvement of other unmeasured cognitive or emotional factors, such as rumination or general emotion dysregulation [47]. These results provide a theoretical and empirical foundation for future development of more complex models going beyond the mediation discussed in the present work.

### Limitations and future directions

Several limitations should be acknowledged in interpreting these findings. First, the sample predominantly consists of WhatsApp users, which may limit the generalizability of results to other social media platforms such as TikTok or Instagram, where usage patterns and FoMO dynamics might differ [33]. Furthermore, excluding individuals who do not use social media or messengers limits our conclusions to active users. Second, the cross-sectional design precludes causal inferences, and unmeasured variables such as socioeconomic status or daily stress levels may confound the observed relationships [48]. Additionally, as all constructs were measured via self-report in a single online session, the potential for common method variance (CMV) cannot be ruled out, which may have inflated the observed bivariate associations. The sample also included minors (aged 13 and older). Because the validity of the present adult-validated measures is not fully established in adolescents, this age inclusion presents a limitation. Furthermore, although we retained the original structure of the FoMO scale to maintain comparability, the low factor loadings of two specific state FoMO items (FoMO5 and FoMO6) indicate potential measurement noise. This psychometric limitation may constrain our interpretive confidence, suggesting that the precise magnitude of the observed mediational pathways should be viewed cautiously. Future research should address these issues by using longitudinal design, objective behavioral logging, cross-cultural samples with age-validated measures, and refined measurement models.

## Conclusion

In conclusion, this study shows that state FoMO is a key mechanism linking both Neuroticism and lower Conscientiousness to PSMU. While state FoMO is not statistically stronger than trait FoMO in the Neuroticism pathway, it is a more consistent mediator across different personality traits. Notably, the association for low Conscientiousness is primarily explained by situational online anxiety (state FoMO) rather than general dispositional tendencies. These findings support the I-PACE model’s distinction between distal predispositions and proximal situational triggers in the development of behavioral addictions [16]. Identifying these specific pathways provides practical directions for designing platform-based environmental interventions and highlights the need for broader emotion-regulation training.

## Supplementary Information

Below is the link to the electronic supplementary material.

Supplementary Material 1

## Data Availability

Data can be obtained via the osf link https://osf.io/f7qwh/ .

## Abbreviations

BFI: Big Five Inventory
FoMO: Fear of Missing Out
I-PACE: Interaction of Person-Affect-Cognition-Execution
PSMU: Problematic Social Media Use
SNS-AT: Social Networking Sites – Addiction Test
