# Supplementary material for: Investigating associations between personality and problematic social media use by considering trait and state FoMO as mediators
Source: BMC Psychol. 2026 Nov 2;14:1582. doi: 10.1186/s40359-026-05527-w (PMC13729315; doi:10.1186/s40359-026-05527-w)
Supplement: Supplementary file 1 [file 40359_2026_5527_MOESM1_ESM.docx]

| **Supplementary Table 1 Generalized linear model mediation analysis with the variables trait/state FoMO (mediators), Neuroticism (predictor), SNS-AT (dependent variable), age (covariates), gender (covariates)** | | | | | | | | |
| --- | --- | --- | --- | --- | --- | --- | --- | --- |
|  | | | | **95% CI** | |  | | |
| **Type** | **Effect** | **Estimate** | **SE** | **Lower** | **Upper** | **β** | **z** | **p** |
| **Indirect** | **Gender ⇒ FoMO_state ⇒ SNS_AT** | -0.37 | 0.22 | -0.79 | 0.06 | -0.03 | -1.69 | 0.09 |
|  | **Gender ⇒ FoMO_trait ⇒ SNS_AT** | 0.02 | 0.05 | -0.09 | 0.12 | 0.00 | 0.33 | 0.74 |
|  | **Neuroticism ⇒ FoMO_state ⇒ SNS_AT** | 0.65 | 0.13 | 0.39 | 0.92 | 0.10 | 4.85 | <.001 |
|  | **Neuroticism ⇒ FoMO_trait ⇒ SNS_AT** | 0.27 | 0.09 | 0.10 | 0.44 | 0.04 | 3.10 | 0.00 |
|  | **Age ⇒ FoMO_state ⇒ SNS_AT** | -0.03 | 0.01 | -0.05 | -0.01 | -0.05 | -2.56 | 0.01 |
|  | **Age ⇒ FoMO_trait ⇒ SNS_AT** | -0.01 | 0.00 | -0.02 | 0.00 | -0.02 | -2.67 | 0.01 |
| **Component** | **Gender ⇒ FoMO_state** | -0.87 | 0.51 | -1.87 | 0.13 | -0.07 | -1.70 | 0.09 |
|  | **FoMO_state ⇒ SNS_AT** | 0.42 | 0.03 | 0.36 | 0.48 | 0.48 | 13.73 | <.001 |
|  | **Gender ⇒ FoMO_trait** | 0.11 | 0.34 | -0.55 | 0.77 | 0.01 | 0.33 | 0.74 |
|  | **FoMO_trait ⇒ SNS_AT** | 0.15 | 0.05 | 0.06 | 0.25 | 0.12 | 3.31 | <.001 |
|  | **Neuroticism ⇒ FoMO_state** | 1.55 | 0.30 | 0.96 | 2.13 | 0.21 | 5.18 | <.001 |
|  | **Neuroticism ⇒ FoMO_trait** | 1.75 | 0.20 | 1.36 | 2.14 | 0.35 | 8.86 | <.001 |
|  | **Age ⇒ FoMO_state** | -0.07 | 0.03 | -0.12 | -0.02 | -0.11 | -2.61 | 0.01 |
|  | **Age ⇒ FoMO_trait** | -0.08 | 0.02 | -0.11 | -0.04 | -0.17 | -4.49 | <.001 |
| **Direct** | **Gender ⇒ SNS_AT** | 0.64 | 0.35 | -0.04 | 1.32 | 0.06 | 1.84 | 0.07 |
|  | **Neuroticism ⇒ SNS_AT** | 1.30 | 0.22 | 0.88 | 1.73 | 0.21 | 6.06 | <.001 |
|  | **Age ⇒ SNS_AT** | -0.06 | 0.02 | -0.09 | -0.02 | -0.10 | -3.17 | 0.00 |
| **Total** | **Gender ⇒ SNS_AT** | 0.29 | 0.42 | -0.54 | 1.11 | 0.03 | 0.68 | 0.50 |
|  | **Neuroticism ⇒ SNS_AT** | 2.23 | 0.25 | 1.74 | 2.71 | 0.35 | 9.04 | <.001 |
|  | **Age ⇒ SNS_AT** | -0.10 | 0.02 | -0.14 | -0.05 | -0.17 | -4.53 | <.001 |

| **Supplementary Table 2 Generalized linear model mediation analysis with the variables trait/state FoMO (mediators), Conscientiousness (predictor), SNS-AT (dependent variable), age (covariates), gender (covariates)** | | | | | | | | |
| --- | --- | --- | --- | --- | --- | --- | --- | --- |
|  | | | | **95% CI** | |  | | |
| **Type** | **Effect** | **Estimate** | **SE** | **Lower** | **Upper** | **β** | **z** | **p** |
| **Indirect** | **Gender ⇒ FoMO_state ⇒ SNS_AT** | -0.09 | 0.22 | -0.51 | 0.34 | -0.01 | -0.40 | 0.69 |
|  | **Gender ⇒ FoMO_trait ⇒ SNS_AT** | 0.17 | 0.09 | -0.01 | 0.34 | 0.02 | 1.89 | 0.06 |
|  | **Age ⇒ FoMO_state ⇒ SNS_AT** | -0.03 | 0.01 | -0.05 | -0.01 | -0.05 | -2.59 | 0.01 |
|  | **Age ⇒ FoMO_trait ⇒ SNS_AT** | -0.02 | 0.01 | -0.03 | -0.01 | -0.03 | -3.39 | <.001 |
|  | **Conscientiousness ⇒ FoMO_state ⇒ SNS_AT** | -0.45 | 0.15 | -0.75 | -0.15 | -0.06 | -2.97 | 0.00 |
|  | **Conscientiousness ⇒ FoMO_trait ⇒ SNS_AT** | -0.10 | 0.06 | -0.22 | 0.02 | -0.01 | -1.66 | 0.10 |
| **Component** | **Gender ⇒ FoMO_state** | -0.21 | 0.51 | -1.21 | 0.80 | -0.02 | -0.40 | 0.69 |
|  | **FoMO_state ⇒ SNS_AT** | 0.42 | 0.03 | 0.36 | 0.48 | 0.48 | 13.45 | <.001 |
|  | **Gender ⇒ FoMO_trait** | 0.73 | 0.36 | 0.03 | 1.42 | 0.08 | 2.04 | 0.04 |
|  | **FoMO_trait ⇒ SNS_AT** | 0.23 | 0.05 | 0.14 | 0.32 | 0.19 | 5.11 | <.001 |
|  | **Age ⇒ FoMO_state** | -0.07 | 0.03 | -0.12 | -0.02 | -0.11 | -2.64 | 0.01 |
|  | **Age ⇒ FoMO_trait** | -0.08 | 0.02 | -0.12 | -0.05 | -0.18 | -4.54 | <.001 |
|  | **Conscientiousness ⇒ FoMO_state** | -1.07 | 0.35 | -1.77 | -0.38 | -0.13 | -3.04 | 0.00 |
|  | **Conscientiousness ⇒ FoMO_trait** | -0.43 | 0.24 | -0.91 | 0.05 | -0.07 | -1.75 | 0.08 |
| **Direct** | **Gender ⇒ SNS_AT** | 1.14 | 0.35 | 0.46 | 1.83 | 0.11 | 3.26 | 0.00 |
|  | **Age ⇒ SNS_AT** | -0.05 | 0.02 | -0.09 | -0.02 | -0.09 | -2.83 | 0.01 |
|  | **Conscientiousness ⇒ SNS_AT** | -0.88 | 0.24 | -1.36 | -0.41 | -0.12 | -3.67 | <.001 |
| **Total** | **Gender ⇒ SNS_AT** | 1.22 | 0.44 | 0.36 | 2.08 | 0.11 | 2.79 | 0.01 |
|  | **Age ⇒ SNS_AT** | -0.10 | 0.02 | -0.14 | -0.06 | -0.18 | -4.46 | <.001 |
|  | **Conscientiousness ⇒ SNS_AT** | -1.44 | 0.30 | -2.03 | -0.85 | -0.19 | -4.79 | <.001 |

| **Supplementary Table 3. CFA results for FoMO Scale**  **Supplementary Table 3a. Factor Loadings** | | | | | | |
| --- | --- | --- | --- | --- | --- | --- |
| **Factor** | **Indicator** | **Estimate** | **SE** | **Z** | **p** | **Stand. Estimate** |
| **trait FoMO** | **FoMO1** | 0.986 | 0.0403 | 24.48 | <.001 | 0.856 |
|  | **FoMO2** | 1.078 | 0.0410 | 26.26 | <.001 | 0.905 |
|  | **FoMO3** | 0.694 | 0.0489 | 14.18 | <.001 | 0.576 |
|  | **FoMO4** | 0.541 | 0.0480 | 11.27 | <.001 | 0.476 |
|  | **FoMO5** | 0.185 | 0.0508 | 3.63 | <.001 | 0.161 |
| **state FoMO** | **FoMO6** | 0.117 | 0.0423 | 2.76 | 0.006 | 0.124 |
|  | **FoMO7** | 0.570 | 0.0480 | 11.86 | <.001 | 0.501 |
|  | **FoMO8** | 0.881 | 0.0428 | 20.58 | <.001 | 0.778 |
|  | **FoMO9** | 0.783 | 0.0469 | 16.72 | <.001 | 0.666 |
|  | **FoMO10** | 0.909 | 0.0432 | 21.05 | <.001 | 0.791 |
|  | **FoMO11** | 0.948 | 0.0473 | 20.04 | <.001 | 0.765 |
|  | **FoMO12** | 0.550 | 0.0553 | 9.95 | <.001 | 0.426 |

| **Supplementary Table 3b. Factor Covariances** | | | | | | |
| --- | --- | --- | --- | --- | --- | --- |
|  |  | **Estimate** | **SE** | **Z** | **p** | **Stand. Estimate** |
| **trait FoMO** | **trait FoMO** | 1.000ᵃ |  |  |  |  |
|  | **state FoMO** | 0.451 | 0.0405 | 11.2 | <.001 | 0.451 |
| **state FoMO** | **state FoMO** | 1.000ᵃ |  |  |  |  |
| ᵃ fixed parameter | | | | | | |

| **Supplementary Table 3c. Test for Exact Fit** | | |
| --- | --- | --- |
| **χ²** | **df** | **p** |
| 543 | 53 | <.001 |

| **Supplementary Table 3d. Fit Measures** | | | | |
| --- | --- | --- | --- | --- |
|  | | | **RMSEA 90% CI** | |
| **CFI** | **TLI** | **RMSEA** | **Lower** | **Upper** |
| 0.809 | 0.762 | 0.127 | 0.117 | 0.136 |

**Supplementary Table 3e. Construct Reliability and Discriminant Validity**

| Construct | CR | AVE | trait FoMO | state FoMO |
| --- | --- | --- | --- | --- |
| trait FoMO | 0.83 | 0.628 | 0.792 |  |
| state FoMO | 0.784 | 0.329 | 0.451 | 0.573 |

| **Supplementary Table 4. Spearman Correlation Matrix for whole cohort** | | | | | | | | | |
| --- | --- | --- | --- | --- | --- | --- | --- | --- | --- |
|  |  | **Extraversion** | **Neuroticism** | **Agreeableness** | **Openness** | **Conscientiousness** | **FoMO_trait** | **FoMO_state** | **SNS_AT** |
| **Extraversion** | **Spearman's rho** | — |  |  |  |  |  |  |  |
|  | **df** | — |  |  |  |  |  |  |  |
|  | **p-value** | — |  |  |  |  |  |  |  |
| **Neuroticism** | **Spearman's rho** | -0.332*** | — |  |  |  |  |  |  |
|  | **df** | 574 | — |  |  |  |  |  |  |
|  | **p-value** | <.001 | — |  |  |  |  |  |  |
| **Agreeableness** | **Spearman's rho** | 0.103* | -0.230*** | — |  |  |  |  |  |
|  | **df** | 574 | 574 | — |  |  |  |  |  |
|  | **p-value** | 0.014 | <.001 | — |  |  |  |  |  |
| **Openness** | **Spearman's rho** | 0.231*** | -0.029 | 0.196*** | — |  |  |  |  |
|  | **df** | 574 | 574 | 574 | — |  |  |  |  |
|  | **p-value** | <.001 | 0.481 | <.001 | — |  |  |  |  |
| **Conscientiousness** | **Spearman's rho** | 0.186*** | -0.203*** | 0.219*** | 0.057 | — |  |  |  |
|  | **df** | 574 | 574 | 574 | 574 | — |  |  |  |
|  | **p-value** | <.001 | <.001 | <.001 | 0.174 | — |  |  |  |
| **FoMO_trait** | **Spearman's rho** | -0.168*** | 0.346*** | -0.062 | -0.069 | -0.056 | — |  |  |
|  | **df** | 574 | 574 | 574 | 574 | 574 | — |  |  |
|  | **p-value** | <.001 | <.001 | 0.137 | 0.100 | 0.179 | — |  |  |
| **FoMO_state** | **Spearman's rho** | -0.050 | 0.177*** | -0.080 | -0.128** | -0.107** | 0.436*** | — |  |
|  | **df** | 574 | 574 | 574 | 574 | 574 | 574 | — |  |
|  | **p-value** | 0.229 | <.001 | 0.056 | 0.002 | 0.010 | <.001 | — |  |
| **SNS_AT** | **Spearman's rho** | -0.158*** | 0.344*** | -0.038 | -0.070 | -0.158*** | 0.443*** | 0.574*** | — |
|  | **df** | 574 | 574 | 574 | 574 | 574 | 574 | 574 | — |
|  | **p-value** | <.001 | <.001 | 0.363 | 0.095 | <.001 | <.001 | <.001 | — |
| Note. * p < .05, ** p < .01, *** p < .001 | | | | | | | | | |

| **Supplementary Table 5a. Pearson Correlation Matrix for adult cohort** | | | | | | | | | |
| --- | --- | --- | --- | --- | --- | --- | --- | --- | --- |
|  |  | **Extraversion** | **Agreeableness** | **Neuroticism** | **Openness** | **Conscientiousness** | **FoMO_trait** | **FoMO_state** | **SNS_AT** |
| **Extraversion** | **Pearson's r** | — |  |  |  |  |  |  |  |
|  | **df** | — |  |  |  |  |  |  |  |
|  | **p-value** | — |  |  |  |  |  |  |  |
| **Agreeableness** | **Pearson's r** | 0.098* | — |  |  |  |  |  |  |
|  | **df** | 480 | — |  |  |  |  |  |  |
|  | **p-value** | 0.031 | — |  |  |  |  |  |  |
| **Neuroticism** | **Pearson's r** | -0.351*** | -0.277*** | — |  |  |  |  |  |
|  | **df** | 480 | 480 | — |  |  |  |  |  |
|  | **p-value** | <.001 | <.001 | — |  |  |  |  |  |
| **Openness** | **Pearson's r** | 0.240*** | 0.188*** | -0.071 | — |  |  |  |  |
|  | **df** | 480 | 480 | 480 | — |  |  |  |  |
|  | **p-value** | <.001 | <.001 | 0.122 | — |  |  |  |  |
| **Conscientiousness** | **Pearson's r** | 0.202*** | 0.204*** | -0.272*** | 0.059 | — |  |  |  |
|  | **df** | 480 | 480 | 480 | 480 | — |  |  |  |
|  | **p-value** | <.001 | <.001 | <.001 | 0.193 | — |  |  |  |
| **FoMO_trait** | **Pearson's r** | -0.153*** | -0.093* | 0.354*** | -0.107* | -0.104* | — |  |  |
|  | **df** | 480 | 480 | 480 | 480 | 480 | — |  |  |
|  | **p-value** | <.001 | 0.042 | <.001 | 0.019 | 0.023 | — |  |  |
| **FoMO_state** | **Pearson's r** | -0.048 | -0.085 | 0.224*** | -0.088 | -0.158*** | 0.463*** | — |  |
|  | **df** | 480 | 480 | 480 | 480 | 480 | 480 | — |  |
|  | **p-value** | 0.295 | 0.063 | <.001 | 0.052 | <.001 | <.001 | — |  |
| **SNS_AT** | **Pearson's r** | -0.146** | -0.027 | 0.374*** | -0.055 | -0.230*** | 0.437*** | 0.582*** | — |
|  | **df** | 480 | 480 | 480 | 480 | 480 | 480 | 480 | — |
|  | **p-value** | 0.001 | 0.549 | <.001 | 0.225 | <.001 | <.001 | <.001 | — |
| Note. * p < .05, ** p < .01, *** p < .001 | | | | | | | | | |

| **Supplementary Table 5b. Spearman Correlation Matrix for adult cohort** | | | | | | | | | |
| --- | --- | --- | --- | --- | --- | --- | --- | --- | --- |
|  |  | **Extraversion** | **Agreeableness** | **Neuroticism** | **Openness** | **Conscientiousness** | **FoMO_trait** | **FoMO_state** | **SNS_AT** |
| **Extraversion** | **Spearman's rho** | — |  |  |  |  |  |  |  |
|  | **df** | — |  |  |  |  |  |  |  |
|  | **p-value** | — |  |  |  |  |  |  |  |
| **Agreeableness** | **Spearman's rho** | 0.091* | — |  |  |  |  |  |  |
|  | **df** | 480 | — |  |  |  |  |  |  |
|  | **p-value** | 0.046 | — |  |  |  |  |  |  |
| **Neuroticism** | **Spearman's rho** | -0.334*** | -0.278*** | — |  |  |  |  |  |
|  | **df** | 480 | 480 | — |  |  |  |  |  |
|  | **p-value** | <.001 | <.001 | — |  |  |  |  |  |
| **Openness** | **Spearman's rho** | 0.225*** | 0.125** | -0.049 | — |  |  |  |  |
|  | **df** | 480 | 480 | 480 | — |  |  |  |  |
|  | **p-value** | <.001 | 0.006 | 0.280 | — |  |  |  |  |
| **Conscientiousness** | **Spearman's rho** | 0.195*** | 0.215*** | -0.253*** | 0.041 | — |  |  |  |
|  | **df** | 480 | 480 | 480 | 480 | — |  |  |  |
|  | **p-value** | <.001 | <.001 | <.001 | 0.365 | — |  |  |  |
| **FoMO_trait** | **Spearman's rho** | -0.173*** | -0.096* | 0.343*** | -0.107* | -0.094* | — |  |  |
|  | **df** | 480 | 480 | 480 | 480 | 480 | — |  |  |
|  | **p-value** | <.001 | 0.034 | <.001 | 0.019 | 0.040 | — |  |  |
| **FoMO_state** | **Spearman's rho** | -0.053 | -0.053 | 0.195*** | -0.100* | -0.128** | 0.461*** | — |  |
|  | **df** | 480 | 480 | 480 | 480 | 480 | 480 | — |  |
|  | **p-value** | 0.244 | 0.244 | <.001 | 0.028 | 0.005 | <.001 | — |  |
| **SNS_AT** | **Spearman's rho** | -0.152*** | -0.040 | 0.352*** | -0.062 | -0.200*** | 0.452*** | 0.562*** | — |
|  | **df** | 480 | 480 | 480 | 480 | 480 | 480 | 480 | — |
|  | **p-value** | <.001 | 0.377 | <.001 | 0.171 | <.001 | <.001 | <.001 | — |
| Note. * p < .05, ** p < .01, *** p < .001 | | | | | | | | | |

| **Supplementary Table 6 Generalized linear model mediation analysis for Neuroticism pathway on adult cohort** | | | | | | | | |
| --- | --- | --- | --- | --- | --- | --- | --- | --- |
|  | | | | **95% C.I. (a)** | |  | | |
| **Type** | **Effect** | **Estimate** | **SE** | **Lower** | **Upper** | **β** | **z** | **p** |
| **Indirect** | **Neuroticism ⇒ FoMO_trait ⇒ SNS_AT** | 0.321 | 0.0994 | 0.1022 | 0.555 | 0.0510 | 3.23 | 0.001 |
|  | **Neuroticism ⇒ FoMO_state ⇒ SNS_AT** | 0.658 | 0.1418 | 0.3804 | 0.976 | 0.1044 | 4.64 | <.001 |
| **Component** | **Neuroticism ⇒ FoMO_trait** | 1.798 | 0.2164 | 1.3815 | 2.194 | 0.3540 | 8.31 | <.001 |
|  | **FoMO_trait ⇒ SNS_AT** | 0.179 | 0.0509 | 0.0591 | 0.290 | 0.1440 | 3.51 | <.001 |
|  | **Neuroticism ⇒ FoMO_state** | 1.635 | 0.3240 | 0.9791 | 2.294 | 0.2241 | 5.05 | <.001 |
|  | **FoMO_state ⇒ SNS_AT** | 0.402 | 0.0340 | 0.3290 | 0.478 | 0.4661 | 11.83 | <.001 |
| **Direct** | **Neuroticism ⇒ SNS_AT** | 1.375 | 0.2353 | 0.8839 | 1.843 | 0.2181 | 5.84 | <.001 |
| **Total** | **Neuroticism ⇒ SNS_AT** | 2.354 | 0.2665 | 1.8137 | 2.870 | 0.3735 | 8.83 | <.001 |
| Note. Confidence intervals computed with method: Bootstrap percentiles | | | | | | | | |
| Note. Betas are completely standardized effect sizes | | | | | | | | |

| **Supplementary Table 7 Generalized linear model mediation analysis for Conscientiousness pathway on adult cohort** | | | | | | | | |
| --- | --- | --- | --- | --- | --- | --- | --- | --- |
|  | | | | **95% C.I. (a)** | |  | | |
| **Type** | **Effect** | **Estimate** | **SE** | **Lower** | **Upper** | **β** | **z** | **p** |
| **Indirect** | **Conscientiousness ⇒ FoMO_trait ⇒ SNS_AT** | -0.163 | 0.0779 | -0.354 | -0.00844 | -0.0216 | -2.10 | 0.036 |
|  | **Conscientiousness ⇒ FoMO_state ⇒ SNS_AT** | -0.553 | 0.1648 | -0.918 | -0.20172 | -0.0731 | -3.35 | <.001 |
| **Component** | **Conscientiousness ⇒ FoMO_trait** | -0.633 | 0.2759 | -1.217 | -0.03805 | -0.1039 | -2.29 | 0.022 |
|  | **FoMO_trait ⇒ SNS_AT** | 0.258 | 0.0497 | 0.139 | 0.37480 | 0.2082 | 5.19 | <.001 |
|  | **Conscientiousness ⇒ FoMO_state** | -1.379 | 0.3935 | -2.226 | -0.50292 | -0.1577 | -3.51 | <.001 |
|  | **FoMO_state ⇒ SNS_AT** | 0.401 | 0.0349 | 0.321 | 0.47849 | 0.4639 | 11.48 | <.001 |
| **Direct** | **Conscientiousness ⇒ SNS_AT** | -1.022 | 0.2719 | -1.578 | -0.45071 | -0.1353 | -3.76 | <.001 |
| **Total** | **Conscientiousness ⇒ SNS_AT** | -1.738 | 0.3352 | -2.464 | -0.99762 | -0.2300 | -5.18 | <.001 |
| Note. Confidence intervals computed with method: Bootstrap percentiles | | | | | | | | |
| Note. Betas are completely standardized effect sizes | | | | | | | | |

### **Supplementary Figure1. Frequency Plots for age grouped by gender**

##### **Female Male**
